# Progress on Crowding Effect in Cell-like Structures

**DOI:** 10.3390/membranes12060593

**Published:** 2022-06-03

**Authors:** Chao Li, Xiangxiang Zhang, Mingdong Dong, Xiaojun Han

**Affiliations:** 1 State Key Laboratory of Urban Water Resource and Environment, School of Chemistry and Chemical Engineering, Harbin Institute of Technology, 92 West Da-Zhi Street, Harbin 150001, China; 17b925070@stu.hit.edu.cn (C.L.); 19b925073@stu.hit.edu.cn (X.Z.); 2 Interdisciplinary Nanoscience Center (iNANO), Aarhus University, 8000 Aarhus, Denmark

**Keywords:** crowding effect, cell-like structures, actin assembly behavior, tubulin aggregation behavior, gene expression

## Abstract

Several biological macromolecules, such as proteins, nucleic acids, and polysaccharides, occupy about 30% of the space in cells, resulting in a crowded macromolecule environment. The crowding effect within cells exerts an impact on the functions of biological components, the assembly behavior of biomacromolecules, and the thermodynamics and kinetics of metabolic reactions. Cell-like structures provide confined and independent compartments for studying the working mechanisms of cells, which can be used to study the physiological functions arising from the crowding effect of macromolecules in cells. This article mainly summarizes the progress of research on the macromolecular crowding effects in cell-like structures. It includes the effects of this crowding on actin assembly behavior, tubulin aggregation behavior, and gene expression. The challenges and future trends in this field are presented at the end of the paper.

## 1. Introduction

**Figure 1:**
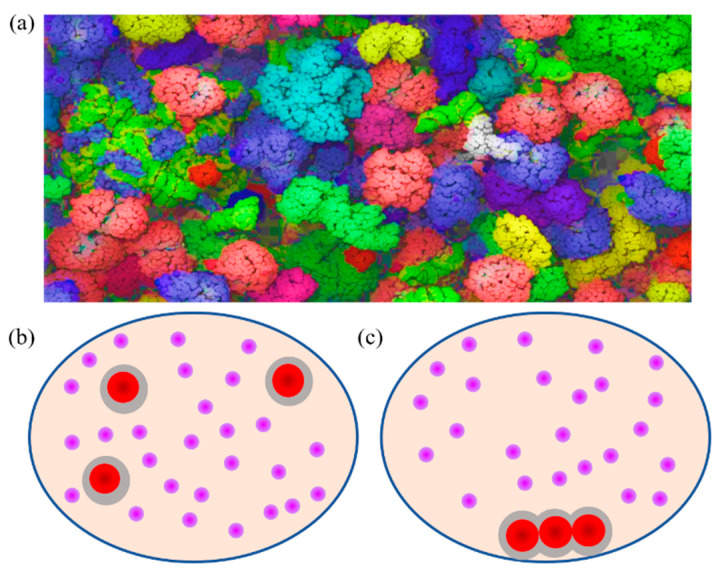
(**a**) Rendering of the *E. coli* cytoplasm model, in which different-colored shapes represent different macromolecules, including GFP, tRNA, etc. Reprinted with permission from Ref. [7]. 2010, McGuffee, Elcock. Crowding effect illustration in a cell-like structure containing large red spheres and small purple spheres before crowding (**b**) and after crowding (**c**).

The cytoplasm is a complex and dense network system composed of proteins, nucleic acids, polysaccharides, lipids, and small molecules, which work together to maintain the basic processes of life [1,2,3]. About 30% of the space in cells is occupied by macromolecules. The concentration of all the macromolecules in the cytoplasm is as high as 80–200 g/L [4,5,6]. Elcock et al. created a visualized cytoplasmic model of Escherichia coli (*E. coli*) (Figure 1a) [7]. The cytoplasm of *E. coli* contains at least 50 different types of macromolecule, reflecting the diversity and complexity of intracellularly crowded macromolecules. All cellular biochemical processes, such as energy supply, gene expression, cell division, etc., take place in this highly crowded, confined space [8,9,10,11]. Therefore, the intracellular macromolecular crowding effect, also known as the excluded volume effect, is an important research field. This phenomenon was proposed by Minton in 1980s [12,13].

The intracellular excluded volume effect results from the presence of molecules of different sizes in cells [14,15]. In the typical model shown in Figure 1b, the large red spheres represent fewer, larger macromolecules in the cell. The purple spheres represent the abundant smaller molecules in the cell. There is no interaction between these two molecules. When larger and smaller molecules are present simultaneously in cells with limited space, there is an excluded volume layer around the large sphere that cannot be reached by the small spheres (gray annular area around the large red sphere) due to the steric effects. The number of small spheres is much larger than that of large spheres, so the small spheres play a major role in contributing to the entropy of the system. The larger spheres tend to aggregate (Figure 1c) to minimize the excluded volume, leading to the maximum amount of space for the small spheres, which consequently maximizes the entropy of the system. This phenomenon is called the crowding effect. The maximization of water entropy also contributes to the crowding effect.

Crowding conditions are created by the addition of macromolecules, such as dextran [16,17,18], seaweed polysaccharide [19], polyethylene glycol [20,21,22], hyaluronic acid [23,24], bovine serum albumin [25,26], cell extracts [27], etc. to the solution, in order to mimic the intracellular macromolecular crowding environment and further to study and explain the biological phenomena in cells. However, these systems are usually developed in solutions with large volume, which is different from cell size. In recent years, the field of cell-like structures has achieved great progresses [28,29,30,31]. The study of the intracellular macromolecular crowding effects of artificial cells has received extensive attention [32,33]. Cell-like structures have a similar volume to real cells and provide enclosed environments. Cell-like structures provide ideal platforms for simulating macromolecular crowding effects in cells [34,35].

This article summarizes the progress of research on the crowding effects in cell-like structures (confined spaces) (Table 1). Different macromolecules create a variety of crowding systems, resulting in diverse physiological functions, including actin assembly behavior, tubulin aggregation behavior, and gene expression.

## 2. Impact of Crowding Media on the Physical and Chemical Properties of Biological Macromolecules

Crowding media have a significant impact on the physical and chemical properties of biological molecules [49]. The aggregation of macromolecules results in an increase in the effective concentrations of functional biological molecules and a decrease in the association energy barrier, which accelerates reaction-limited biochemical processes [15]. However, crowded media act as obstacles to reduce the diffusion rates of biological molecules [50]. Therefore, under crowded conditions, diffusion-limited biochemical processes are decelerated.

Crowded environments can influence the reaction rate dramatically. Popielec et al. found that the crowding agents ficoll and polyethylene glycol (PEG) showed opposite effects on NS3/4A catalytic reaction kinetics: ficoll accelerated the reaction while PEG slowed it down [51]. The authors attributed this unexpected phenomenon to the different kinds of interactions between the protease and these crowders. The ficoll exhibited mainly hard-core repulsion to increase the concentration of substrates, while the PEG reduced the diffusion of both the enzyme and the substrates. Fuentes-Lemus et al. found that the macromolecular environment resulted in a lower available volume and increased the concentration of the reactive species, which increased the oxidation rate of free and peptide tryptophan [52].

The cytoplasm is a liquid containing a dense assemblage of proteins. When the cytoplasm is overcrowded, the viscosity increases significantly, resulting in a dramatic decrease in their mobility [53]. Miermont et al. found cytoplasmic crowding in a yeast cell due to the reduction in the cell volume by severe osmotic stress [54]. The cytoplasmic crowding caused a decrease in protein mobility and eventually led to the stalling of multiple transduction signals. Golkaram et al. investigated the mobility of biological tracer molecules in a dextran crowding environment [55]. The transcription factor mobility decreased obviously with the increasing fractions of the crowder volume, which consequently reduced the noise of the gene expression due to the increasing contact time of the transcription factor with the promoter. Junker et al. found that the mobility of larger tracer molecules was reduced in a more pronounced way in crowding conditions with PEG or ficoll polymers due to the molecular sieving effect [56].

## 3. Crowding Effects Induced Actin Assembly Behavior in Confined Spaces

**Figure 2:**
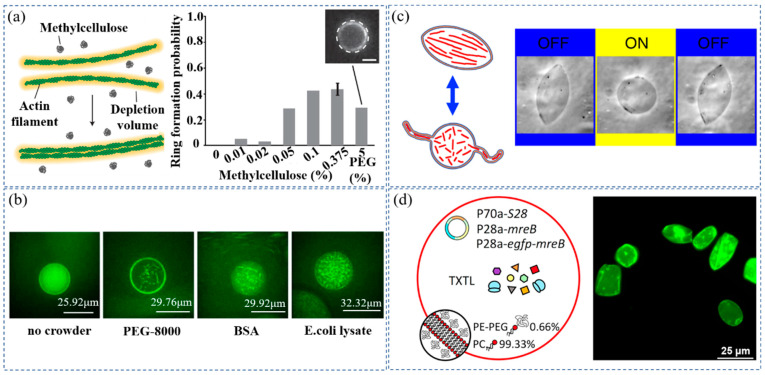
(**a**) The crowding effect of macromolecules promoted actin-ring formation at the equatorial plane of the droplet. Reprinted with permission from Ref. [36] 2015, Springer Nature. (**b**) FtsZ bundle formation with no crowder, polymeric crowder PEG-8000, protein crowder BSA, and *E. coli* lysate. Reprinted with permission from Ref. [37] 2015, American Chemical Society. (**c**) Repeated deformation of spindle-shaped liposomes encapsulating high-concentration actin. Reprinted with permission from Ref. [38] 2018, Springer Nature. (**d**) MreB produced by cell-free expression deformed liposomes with membranes composed of PC and PE-PEG. Reprinted with permission from Ref. [39] 2020, American Chemical Society.

The cytoskeleton controls the contraction of cells and participates in various functional activities, such as cell division, deformation, movement, and migration [57,58]. The extracellular assembly conditions and influencing factors of cytoskeleton proteins, such as actin, have deeply been investigated [59,60,61,62]. The cytoskeleton proteins were encapsulated in cell-like structures to study their assembly behavior in a confined space [63,64,65,66]. The crowded environments were created in cell-like structures to study the assembly behavior of the cytoskeleton [36,37,38]. As shown in Figure 2a, Miyazaki et al. used methylcellulose as a macromolecular crowder to study the polymerization behavior of actins [36]. The actin monomers were encapsulated in water droplets protected by monolayer phospholipid membrane together with methylcellulose. In the presence of the methylcellulose, the actin filaments aggregated into thick and long actin bundles distributed along the equator of the inner membrane of the droplet due to the crowding effect. In this study, the actin filaments and methylcellulose molecules played the role of large red balls and small purple balls, respectively (Figure 1b,c). The actin filaments (large red balls) contacted each other to release the free volume for methylcellulose molecules (small purple balls). The total free energy of the system decreased due to the increased entropy of the crowder. Similarly, Groen et al. encapsulated a variety of macromolecules in droplets and observed the crowding effect on FtsZ aggregation [37]. They found that the FtsZ bundles were formed by adding the polymeric crowder PEG-8000, the protein-based crowder BSA, and the *E. coli* lysate to the FtsZ polymerization buffer solution (Figure 2b).

Tanaka et al. encapsulated actins in giant unillamellar vesicles (GUVs) to construct artificial cells containing a cytoskeleton [38]. By increasing the concentration of actins in the GUVs, a crowded environment was created in the artificial cells, which was similar to real cells. As shown in Figure 2c, the morphology of the GUVs containing actin filaments changed from spherical to spindle when the crowded environment was formed by the high actin concentration. Under laser irradiation, actin filaments were destroyed, leading to the recovery of the GUVs’ spherical shapes. This process can be repeated multiple times, further deepening our understanding of the mechanisms of cell motility.

Since the cell membrane also contains a large number of diverse proteins and polysaccharides, the crowding of macromolecules occurs not only inside cells, but also on the cell membrane. Garenne et al. expressed the *E. coli* gene mreB inside GUVs composed of egg PC and lipid-PEG [39]. The crowded environments created by PEG molecules on phospholipid bilayer membranes is enough to promote the polymerization of the protein MreB at the inner membrane into a sturdy cytoskeleton capable of transforming spherical liposomes into elongated shapes, such as rod-like compartments (Figure 2d). The macromolecular crowding effect on the cell membrane has a significant impact on cell morphology and behavior. The study of the crowding phenomenon at the cell membrane provides the basis for cellular asymmetry and self-reproduction.

## 4. Aggregation Behavior of Tubulin in a Crowded Environment

**Figure 3:**
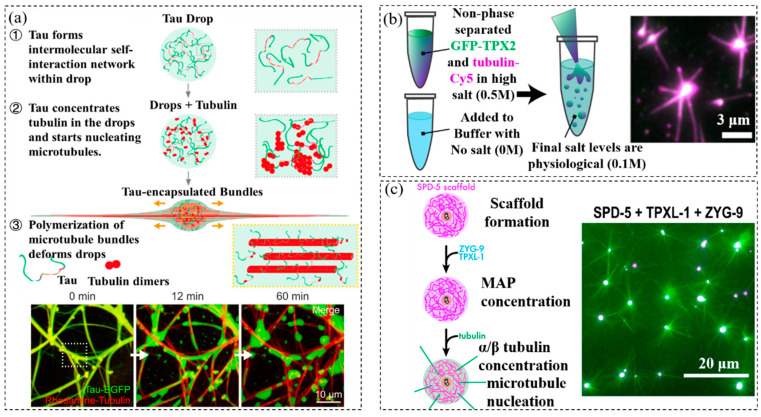
(**a**) Tubulin polymerization inside Tau droplets. Reprinted with permission from Ref. [41] 2018, Elsevier. (**b**) Tubulins nucleation inside TPX2 droplets to allow the formation of microtubules. Reprinted with permission from Ref. [42] 2020, Springer Nature. (**c**) Tubulin nucleation inside SPD-5/TPXL-1/ZYG-9 condensates. Reprinted with permission from Ref. [43] 2017, Elsevier.

In addition to adding macromolecules to create crowded environments in cell-like structures, phase separation is another common method to create crowded environments [67,68,69,70]. Many intrinsically disordered proteins can form liquid-like drops by phase separation [71]. Tau is one of the disordered proteins that can undergo phase separation in crowded environments to form protein droplets [72]. Tau is an important component of the microtubule cytoskeleton in neurons. In healthy brains, Tau localizes to the axons of neurons and consists of four tubulin binding domains surrounded by intrinsically disordered regions. Hyman et al. observed Tau droplet formation using dextran, polyethylene glycol, or ficoll as crowders [41]. Due to the crowding effect, the increase in the crowder entropy led to the formation of enriched phases. The tubulin partitioned into these drops to efficiently increase its concentration. The concentration inside the droplets was more than 10-fold higher than the concentration outside the droplets (Figure 3a). Microtubule bundles polymerized within the droplets to deform them into rod-like shapes. This study used the macromolecular crowding effect to generate phase-separated protein droplets. Tubulin becomes concentrated inside Tau drops, allowing microtubule nucleation inside Tau droplets. These findings have potential implications for Tau function in axonal projections.

TPX2 is an important protein in microtubule nucleation in spindles [73]. The intrinsically disordered properties of TPX2 enable its phase separation in crowded environments. As shown in Figure 3b, King et al. regulated the phase separation of TPX2 protein by adjusting the salt ion concentration in the solution [42]. The TPX2 was maintained in a high-salt (0.5 M) buffer without phase separation. When the TPX2 solution was mixed with water to decrease the concentration of the physiological salt levels (0.1 M), it underwent phase separation to form a co-condensate with tubulins. The tubulins were concentrated inside TPX2 droplets. The phase separation of TPX2 caused the tenfold improvement in the tubulin nucleation efficiency. This study demonstrated how regulated phase separation simultaneously enhances reaction efficiency and spatially coordinate microtubule nucleation, which might facilitate rapid and accurate spindle formation.

Centrosomes are non-membranous compartments in cells [74]. They consist of nanometer-scale centrioles surrounded by a micron-scale, dynamic assembly of protein called the pericentriolar material (PCM). To study how PCM forms spherical compartments that nucleate microtubules, Woodruff et al. reconstituted PCM-dependent microtubule nucleation in vitro (Figure 3c). SPD-5 is a key scaffold protein in dynamic protein assemblies [43]. The macromolecular crowding effect drives the assembly of the SPD-5 into spherical condensates that morphologically and dynamically resemble PCM in vivo. These SPD-5 condensates recruited the microtubule polymerase ZYG-9 and the microtubule-stabilizing protein TPXL-1. These three proteins concentrated the tubulin about four-fold over the background, which was sufficient to cause the nucleation of microtubule asters in vitro.

The crowded environment caused by phase separation has a significant impact on the enrichment and assembly behavior of tubulins. It provides an important method for studying the morphologies and working mechanisms of organelles and membrane-free areas inside cells.

## 5. Crowding Effect on Gene Expression

**Figure 4:**
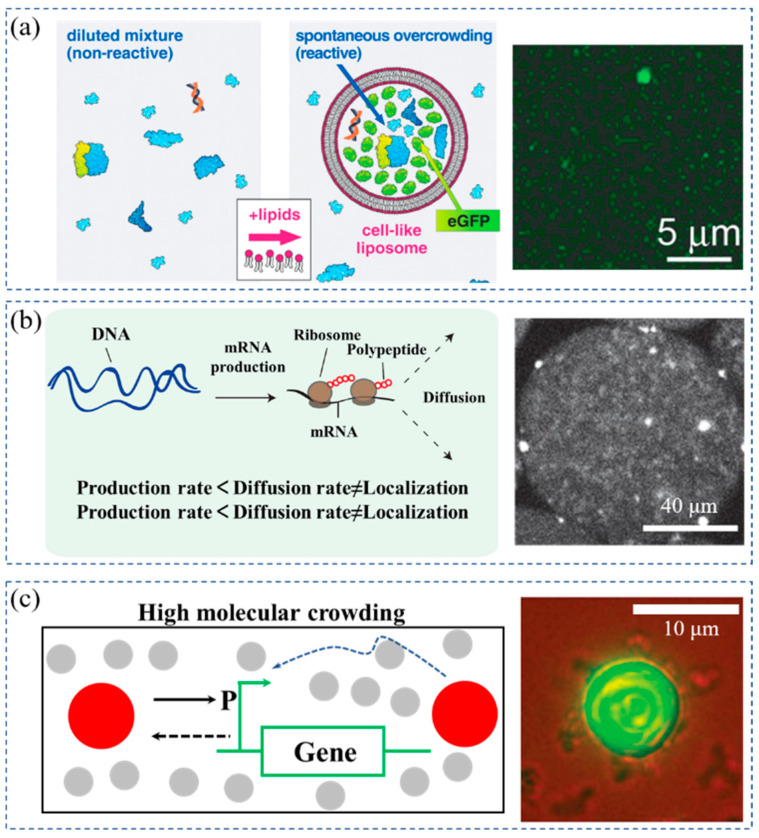
(**a**) A spontaneous concentration of all components of the mixture inside vesicles by spontaneous formation of liposomes, enabling protein synthesis. Reprinted with permission from Ref. [45] 2013, John Wiley and Sons. (**b**) Inhomogeneous distribution of mRNA over one droplet at high ficoll concentrations. Reprinted with permission from Ref. [46] 2015, Springer Nature. (**c**) Enhancement of gene expression in artificial cells by crowding effect. Reprinted with permission from Ref. [47] 2013, Springer Nature.

Cell-free protein synthesis is a rapid and high-throughput expression technology of target proteins. The exogenous DNA or mRNA is used as a template to achieve in vitro protein synthesis by supplementing substrates and energy substances [75,76,77,78]. This technique was used for studying the effects of crowding on gene expression. These multimolecular systems contain a total of about 80 different macromolecular components, including RNA polymerase, ribosomes, tRNAs, translation factors, related enzymes, etc. Stano et al. found that cell-free transcription and translation cannot be achieved at low concentrations of biomacromolecules [45]. However, when the phospholipids were added to the diluted transcription-translation solution, typical images of the vesicles were obtained (shown in Figure 4a). Green fluorescent vesicles were clearly visible against a dark background, which indicated that GFP was effectively produced inside the vesicles. This was possibly due to the formation of crowded vesicles during the assembly process, which resulted from the increase in the substrate concentration. Crowding may provide a suitable environment for the expression of genes in primitive cells at the origin of life.

The crowding effect significantly affects mass transport during transcription and translation [79,80,81]. Hansen et al. encapsulated a cell-free protein synthesis system with macromolecular crowders in droplets and presented a robust method to quantify gene expression noise in vitro [46]. They investigated the changes in noise in the cell-free gene expression of two genes compartmentalized within droplets as a function of DNA copy number and macromolecular crowding. They found that the crowded environment caused protein inhomogeneity and significantly increased noise (Figure 4b). The decreased diffusion caused by the crowded environment led to the spontaneous formation of heterogeneous mRNA microenvironments as local production rates exceeded the diffusion rates of the macromolecules. This heterogeneity directly increased the system’s noise. The crowding effect causes inhomogeneity within the cell space and has a huge impact on mass transport during transcription and translation.

Internal crowded environments are key features of cells throughout evolution, which is essential in maintaining robust gene expression. Tan et al. investigated the effect of crowding on gene expression robustness (Figure 4c) [47]. They created two parameter sets using the same mathematical model. One represented a low crowding environment, while the other represented a high crowding environment. They demonstrated that the highly crowded conditions increased the robustness of the gene expression, which was consistent with their experimental results. In cell-like structures, crowded environments can increase the robustness of gene expression. The crowding effect plays an important role in regulating system dynamics. Therefore, the effect of molecular crowding should be considered when constructing artificial cells.

## 6. Summary and Prospects

This article summarized the progress of research on crowding effects and their consequences in cell-like structures, including actin assembly behavior, tubulin aggregation behavior, and gene expression. The crowding effect exerts a significant impact on the physical properties and functions of cells. Although progress has been achieved using cell-like structures to simulate crowding effects, the field is still in its infancy. Using cell-like structures to closely study the impact of macromolecular crowding on the physiological activities of cells, future research may focus on the following three aspects. First, the lumen of cell-like structures is closer to the real internal environments of cells. Biomimetic microenvironments based on multiple materials have been constructed for cell culture and tissue engineering in vitro [82]. The composition of microenvironments was gradually upgraded from simple macromolecular crowding agents, such as PEG and ficolls, to biological materials that are closer to the real environments of cells, such as collagen and BSA. Macromolecules were used to investigate the effect of crowding on extracellular matrix deposition [23,83], cell differentiation [84], and cancer cell dissemination [85]. The contents of cell-like structures can comprise of real biological molecules. For example, the cytoplasmic matrix of cells, or a solution with similar composition, can serve as the lumen solution for cell-like structures. Second, more complex physiological functions can be studied in the crowded environments of cell-like structures, including the regulation of cellular homeostasis, the generation and action of stress granules, etc. Third, a large number of proteins and polysaccharides can be inserted into the cell membrane, creating a highly crowded environment. The diffusion, aggregation, and configuration of proteins are expected to be studied in crowded membranes [86]. There are two ways to mimic crowded membranes. Different proteins could be conjugated to synthetic lipids via specific interactions, e.g., polyhistidine and Ni^2+^-NTA [87] or streptavidin and biotin [88]. Alternatively, PEG-modified lipid molecules can be used to create crowded environments in the membranes [89]. Natural biological membranes, such as isolated cell membranes and organelle membranes, should be involved to reveal more about the effect of crowding on cell membranes. This field offers great promise for improving our understanding of working cellular mechanisms.

## Figures and Tables

**Table 1:** Progress of research on crowding effect in cell-like structures.

Crowded System	Physiological Function of Crowding Effect	Confined Space	Reference
Methylcellulose, actin	Ring-shaped actin bundles assembled at the inner peripheries	Water-in-oil droplets	[36]
FtsZ, PEG-8000, BSA, *E. coli* lysates	FtsZ bundle formation in microdroplets	Water-in-oil droplets	[37]
Actin	Actin filaments aggregated into thick actin bundles within Giant unillamellar vesicles (GUVs) to deform GUVs into spindle shapes	Giant unillamellar vesicles	[38]
PC, PE-PEG, cell-free protein-synthesis system of MreB	The polymerization of the protein MreB at the inner membrane into a sturdy cytoskeleton capable of transforming spherical GUVs into elongated shapes	Giant unillamellar vesicles	[39]
Endocytosis protein (Epsin1), green fluorescent protein	Epsin1 or GFP were able to drive fission efficiently when bound to the membrane at high coverage	Surface of phospholipid vesicle membrane	[40]
Dextran, polyethylene glycol, ficoll, Tau protein, tubulin	Tubulin partitioned into Tau drops, efficiently increasing tubulin concentration and driving the nucleation of microtubules	Phase-separated protein droplets	[41]
TPX2 protein, tubulin	Phase separation of TPX2 and tubulin could underlie the tenfold improvement in the branching MT nucleation efficiency	Phase-separated protein droplets	[42]
SPD-5 protein, ficoll, dextran, lysozyme	Tubulin was concentrated 4-fold over background to promote tubulin nucleation	Phase-separated protein droplets	[43]
Polyethylene glycol, dextran, actin, long DNA	Actin bundles distributed across the phase interface, deforming the interface and pushing DNA to their ends	Interfacial layer of liquid–liquid phase separation	[44]
Polyethylene glycol, dextran, ELP protein	ELP protein droplets were distributed near the interface between the two phases	Interfacial layer of liquid–liquid phase separation	[9]
Cell-free protein-synthesis system	Green fluorescent protein was expressed	Phospholipid vesicles	[45]
Ficoll, cell-free protein synthesis-system of cyan and yellow fluorescent protein	An order-of-magnitude decrease in the diffusion coefficients of RNA and proteins	Water-in-oil droplets	[46]
Dextran, cell-free protein-synthesis system of GFP	An increase in the robustness of gene expression	Giant unillamellar vesicles	[47]
Ficoll, cell-free protein-synthesis system of GFP	A 10-fold increase in protein noise	Giant unillamellar vesicles	[48]
